# Racial Differences in the Oral Microbiome: Data from Low-Income Populations of African Ancestry and European Ancestry

**DOI:** 10.1128/mSystems.00639-19

**Published:** 2019-11-26

**Authors:** Yaohua Yang, Wei Zheng, Qiuyin Cai, Martha J. Shrubsole, Zhiheng Pei, Robert Brucker, Mark Steinwandel, Seth R. Bordenstein, Zhigang Li, William J. Blot, Xiao-Ou Shu, Jirong Long

**Affiliations:** a Division of Epidemiology, Department of Medicine, Vanderbilt Epidemiology Center, Vanderbilt-Ingram Cancer Center, Vanderbilt University Medical Center, Nashville, Tennessee, USA; b Department of Pathology, New York University School of Medicine, New York, New York, USA; c Rowland Institute, Harvard University, Cambridge, Massachusetts, USA; d International Epidemiology Field Station, Vanderbilt University Medical Center, Rockville, Maryland, USA; e Department of Biological Sciences, Vanderbilt University, Nashville, Tennessee, USA; f Department of Pathology, Microbiology, and Immunology, Vanderbilt University, Nashville, Tennessee, USA; g Department of Biostatistics, University of Florida, Gainesville, Florida, USA; Qingdao Institute of Bioenergy and Bioprocess Technology, Chinese Academy of Sciences

**Keywords:** oral microbiome, racial difference

## Abstract

Increasing evidence indicates the significant racial difference in gut, vaginal, and skin microbiomes. However, little is known regarding the racial difference in the oral microbiome. In this study, deep sequencing of 16S rRNA genes was utilized to assess the oral microbiome in mouth rinse samples of 1,058 African-Americans (AAs) and 558 European-Americans (EAs) from the Southern Community Cohort Study. Generally, AAs had a higher species richness than EAs, with *P = *5.28 × 10^−14^ (Wilcoxon rank sum test) for Faith’s phylogenetic diversity index. A significant difference in overall microbiome composition was observed between AAs and EAs, with *P = *5.94 × 10^−4^ (MiRKAT) for the weighted UniFrac distance matrix. We also found 32 bacterial taxa showing a significant differential abundance or prevalence between the two racial groups at a Bonferroni-corrected *P < *0.05 in linear or logistic regression analyses. Generally, AAs showed a higher abundance of *Bacteroidetes* and a lower abundance of *Actinobacteria* and *Firmicutes*. Interestingly, four periodontal pathogens, Porphyromonas gingivalis, Prevotella intermedia, Treponema denticola, and Filifactor alocis, were more prevalent among AAs than among EAs, with Bonferroni-corrected *P* values of 5.23 × 10^−6^, 4.47 × 10^−6^, 1.08 × 10^−3^, and 4.49 × 10^−5^, respectively. In addition, all of these 32 taxa were significantly correlated with the percentage of genetic African ancestry. These findings call for research to understand how the racial difference in oral microbiome influences the health disparity.

**IMPORTANCE** In this systemic investigation of racial differences in the oral microbiome using a large data set, we disclosed the significant differences in the oral microbial richness/evenness, as well as in the overall microbial composition, between African-Americans and European-Americans. We also found multiple oral bacterial taxa, including several preidentified oral pathogens, showing a significant different abundance or prevalence between African-Americans and European-Americans. Furthermore, these taxa were consistently found to be associated with the percentage of genetic African ancestry. Our findings warrant further research to understand how the racial difference in the oral microbiome influences the health disparity.

## Abstract (precis)

In this systemic investigation of racial differences in the oral microbiome using a large data set, we disclosed the significant differences in the oral microbial richness/evenness, as well as in the overall microbial composition, between African-Americans and European-Americans. We also found multiple oral bacterial taxa, including several preidentified oral pathogens, showing a significant different abundance or prevalence between African-Americans and European-Americans. Furthermore, these taxa were consistently found to be associated with the percentage of genetic African ancestry. Our findings warrant further research to understand how the racial difference in the oral microbiome influences the health disparity.

## INTRODUCTION

It has been estimated that nearly 100 trillion microbes colonize in different human body habitats, collectively composing the microbiota (1). Host-microbiota interactions are deeply involved in various physiological and metabolic activities (2) and hosts’ health (3). The human mouth is heavily colonized by microorganisms (1) and acts as a portal for microbes to gain access to the respiratory and digestive tracts (4). It is well acknowledged that the oral microbiota affects two common oral diseases, dental caries and periodontal disease (5), while recent studies also indicate that the oral microbiota may play roles in maintaining systemic health through nutrition absorption, metabolism, and immune system regulation (4).

Studies have suggested that many host-related factors are associated with diversity and compositions of microbial communities, such as the host’s race/ethnicity (6), genetic background (7), and socioeconomic status (8). Increasing evidence suggests that there are racial differences in microbial profiles (6) of vaginal (9), gut (10), and skin (11) microbiomes. Two studies have investigated the differences in oral microbiome across racial groups (12, 13). In the earlier one (12), two strategies, terminal restriction fragment length polymorphism (t-RFLP) and 16S rRNA gene pyrosequencing, were used to assess microbiota in plaque and saliva samples from 192 individuals of four ethnic affiliations. They found that the oral microbiota of African-Americans (AAs) had lower alpha diversity than that of European-Americans (EAs), Chinese, and Latinos. In the more recent one (13), the 16S rRNA gene was sequenced for the saliva samples of 152 participants from three different climate zones (13). The authors found that both alpha and beta diversity differed significantly among populations from Alaska, Germany, and Africa. However, both studies had a small sample size, and/or a limited number of microorganisms were investigated. Here, we investigated the oral microbiota of 1,616 participants of the Southern Community Cohort Study (SCCS), including 1,058 AAs and 558 EAs.

## RESULTS

### Characteristics of the study participants.

Table 1 shows the general characteristics of the participants included in the present study. A total of 1,616 individuals were involved, including 1,058 AAs and 558 EAs. Overall, the study participants have low social economic status, with ∼57% of AAs and ∼35% of EAs having an annual household income of <$15,000, and ∼32% of AAs and ∼18% of EAs receiving less than 12 years of education. Among the AAs, ∼39% were current smokers and ∼25% were former smokers, while among EAs, ∼32% were current smokers and ∼38% were former smokers. The AAs had worse oral health status than the EAs, with ∼87% versus ∼75% having lost at least one tooth. Among these lifestyle factors, race, age, alcohol drinking, tooth loss, annual household income, and sequencing batch were associated with Faith’s phylogenetic diversity (PD) index, i.e., the overall phylogenetic richness and evenness of oral microbiota (*P < *0.05; see Fig. S1 in the supplemental material).

**TABLE 1:** Characteristics of the participants in the Southern Community Cohort Study

Characteristic	African-Americans (*n* = 1,058)	European-Americans (*n* = 558)
Age (yr), mean ± SD	54.72 ± 8.73	57.95 ± 8.41
Sex, no. (%)		
Female	549 (51.89)	232 (41.58)
Male	509 (48.11)	326 (58.42)
BMI (kg/m^2^), mean ± SD	29.76 ± 7.35	28.53 ± 6.79
Genetic African ancestry (%), mean ± SD	87.39 ± 10.50	0.27 ± 0.75
Annual household income ($), no. (%)		
<15,000	593 (56.69)	192 (35.23)
15,000–24,999	216 (20.65)	74 (13.58)
25,000–49,999	150 (14.34)	106 (19.45)
≥50,000	87 (8.32)	173 (31.74)
Highest level of education, no. (%)		
<High school	335 (31.72)	99 (17.74)
High/vocational school	394 (37.31)	175 (31.36)
Some college	191 (18.09)	110 (19.71)
≥College	136 (12.88)	174 (31.18)
Alcohol consumption, no. (%)^*a*^		
None	492 (47.40)	252 (46.32)
Light	295 (28.42)	179 (32.90)
Moderate	120 (11.56)	60 (11.03)
Heavy	131 (12.62)	53 (9.74)
Tobacco smoking, no. (%)		
Never	381 (36.01)	166 (29.75)
Current	414 (39.13)	178 (31.90)
Former	263 (24.86)	214 (38.35)
Tooth loss, no. (%) of participants		
None	85 (13.08)	104 (25.12)
1–10	316 (48.62)	182 (43.96)
>10, not all	149 (22.92)	65 (15.70)
All	100 (15.38)	63 (15.22)

a Light, <1 drink/day; moderate, 1 to 2 drinks/day; heavy, >2 drinks/day.

10.1128/mSystems.00639-19.1 FIG S1 Effect sizes and standard errors of other factors on Faith’s phylogenetic diversity index in combined analyses and in stratified analyses by the two sequencing batches. Download FIG S1, PDF file, 0.5 MB. Copyright © 2019 Yang et al. 2019 Yang et al. This content is distributed under the terms of the Creative Commons Attribution 4.0 International license.

### Difference of overall microbial diversity and composition between AAs and EAs.

**FIG 1:**
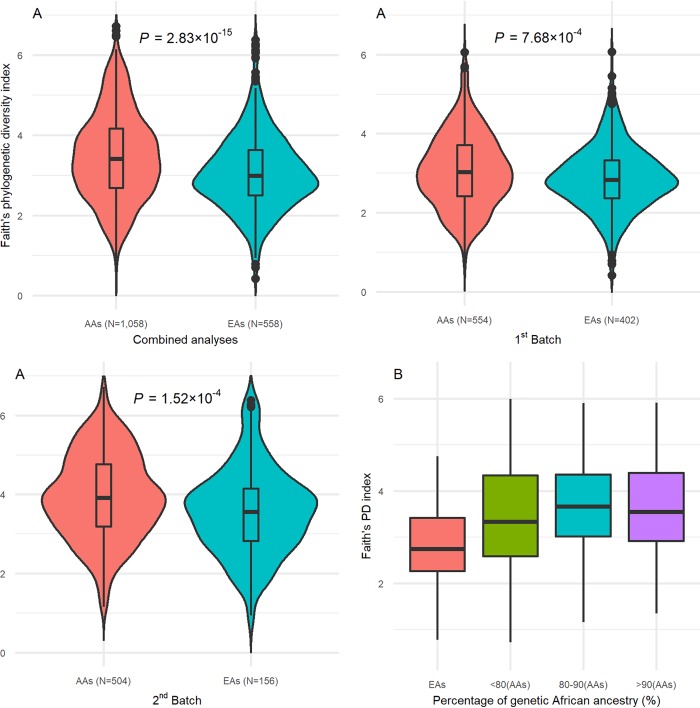
Significant differences of oral microbial richness and evenness between AAs and EAs. The oral microbial richness and evenness were estimated using Faith’s phylogenetic diversity (PD) index. *P* values were calculated using Wilcoxon rank sum test. (A) Significant differences of Faith’s PD index were consistently observed in combined analyses and in stratified analyses by the two sequencing batches. (B) Increases in Faith’s PD index along with the increase in hosts’ genetic African ancestry.

**FIG 2:**
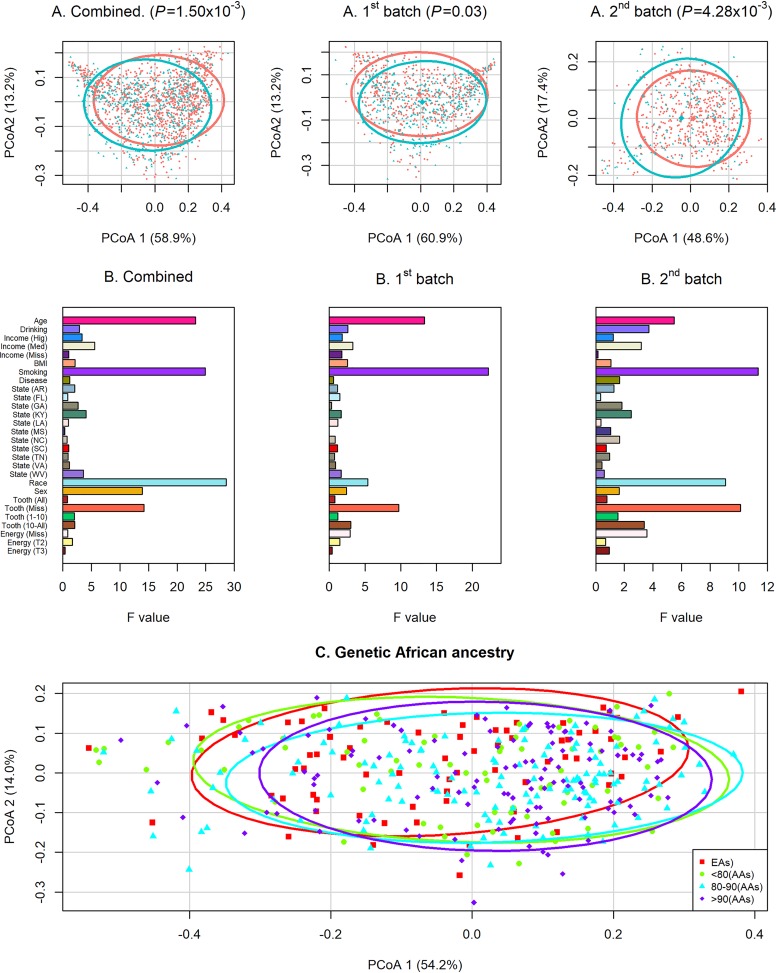
Significant differences of overall oral microbiome composition between AAs and EAs. The overall oral microbiome composition was estimated using the weighted UniFrac matrices, and the *P* values were calculated using MiRKAT. (A) Red circles and blue triangles represent AAs and EAs, respectively. The ellipses and centroids (diamond) of the first two principal-coordinate analyses of weighted UniFrac distances for AAs (red) and EAs (blue) were estimated using the “dataEllipse” function of the R package “car.” (B) *F* values of other factors on weighted UniFrac distance in combined analyses and in stratified analyses by the two sequencing batches. (C) Changes in the first two principal coordinates of weighted UniFrac distance along with the increase in hosts’ genetic African ancestry.

The data collection of the present study represented well for both alpha and beta diversity in both AAs and EAs. As shown in Fig. S2, within both AAs and EAs, Faith’s PD index (Fig. S2A and B) and weighted UniFrac distance (Fig. S2C and D) decreased along with the increase in samples size. In addition, when the sample size of AAs reached ∼800 (<final sample size of 1,058) and the sample size of EAs reached ∼450 (<final sample size of 558), both Faith’s PD index and weighted UniFrac distance did not change materially. In the present study, AAs showed higher alpha diversity, i.e., microbial richness and evenness, than EAs, with a *P* value of 2.83 × 10^−15^ for Faith’s PD index, as estimated by Wilcoxon rank sum test (Fig. 1A). The significantly higher Faith PD index among AAs than among EAs was consistently observed in the two sequencing batches, with *P* values (Wilcoxon rank sum test) of 7.68 × 10^−4^ and 1.52 × 10^−4^ for the first and the second batch, respectively (Fig. 1A). Along with the increase in hosts’ percentage of genetic African ancestry, the Faith PD index of oral microbiome increased (Fig. 1B). A significant difference in beta diversity was also found between AAs and EAs (Fig. 2A) with *P* values of 1.50 × 10^−3^, 1.17 × 10^−7^, and 3.16 × 10^−5^ for the weighted UniFrac distance, unweighted UniFrac distance, and Bray-Curtis dissimilarity matrices, respectively, as tested by MiRKAT. The significant differences in beta diversity were also consistent between the two sequencing batches (Fig. 2A), with *P* values (MiRKAT), for the weighted UniFrac distance, unweighted UniFrac distance, and Bray-Curtis dissimilarity matrices of 0.03, 1.91 × 10^−4^, and 8.88 × 10^−4^ for the first batch, and 4.28 × 10^−3^, 2.50 × 10^−6^, and 0.01 for the second batch, respectively. Figure 2B showed the *F* values of other factors on the weighted UniFrac distances in combined analyses and in stratified analyses by the two sequencing batches. In addition, beta diversity was significantly associated with hosts’ percentage of genetic African ancestry, with *P* values (MiRKAT) of 1.52 × 10^−4^ for weighted UniFrac distance, <2.20 × 10^−16^ for unweighted UniFrac distance, and 2.32 × 10^−4^ for Bray-Curtis dissimilarity distance. The changes in weighted UniFrac distance along with the increase in hosts’ percentage of genetic African ancestry are shown in Fig. 2C.

10.1128/mSystems.00639-19.2 FIG S2 Decreases in Faith’s phylogenetic diversity index and weighted UniFrac distance along with the increases in sample size among AAs and EAs. Download FIG S2, PDF file, 0.2 MB. Copyright © 2019 Yang et al. 2019 Yang et al. This content is distributed under the terms of the Creative Commons Attribution 4.0 International license.

### Common bacterial taxa with a significant differential abundance between AAs and EAs.

**FIG 3:**
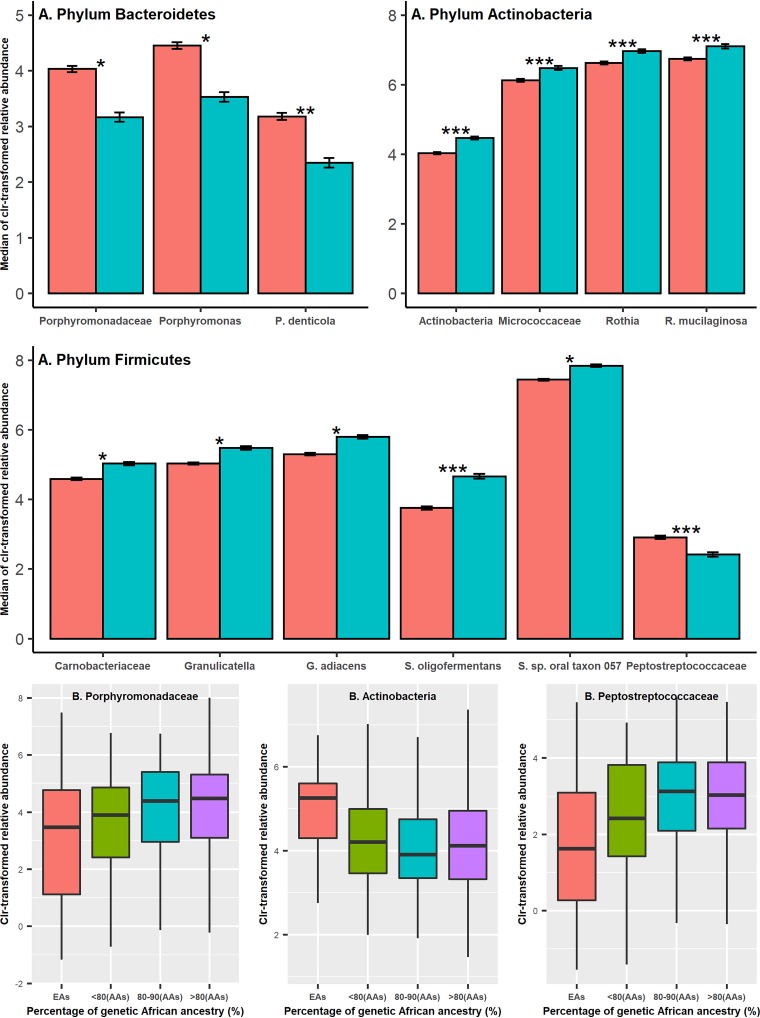
Thirteen common bacterial taxa showing a significant differential abundance between AAs and EAs in linear regression analyses. (A) The associations of taxon abundance and race were evaluated using linear regression analyses. *, **, and *** represent the Bonferroni-corrected *P* values <0.05, <0.01, and <0.001, respectively. (B) Changes in centered-log-ratio-transformed relative abundance of three common taxa along with the increase in hosts’ genetic African ancestry. Similar plots for other common taxa shown in Table 2 are presented in Fig. S4.

Table 2 and Fig. 3A present the 13 taxa with their relative abundances showing a significant racial difference in linear regression analyses after Bonferroni correction. Generally, AAs showed a higher abundance of *Bacteroidetes* but a lower abundance of *Actinobacteria* and *Firmicutes* than EAs. In *Bacteroidetes*, three taxa, including *Porphyromonadaceae*, *Porphyromonas*, and Prevotella denticola, showed a significantly higher relative abundance among AAs, with Bonferroni-corrected *P* values of 0.03, 0.05, and 1.56 × 10^−3^, respectively. The phylum *Actinobacteria*, along with three taxa within it, *Micrococcaceae*, *Rothia*, and Rothia mucilaginosa, were found to be significantly more abundant among EAs, with Bonferroni-corrected *P* values of 6.14 × 10^−8^, 1.81 × 10^−6^, 1.14 × 10^−6^, and 1.21 × 10^−5^, respectively. In *Firmicutes*, five taxa, *Carnobacteriaceae*, *Granulicatella*, Granulicatella adiacens, Streptococcus oligofermentans, and *Streptococcus* sp. oral taxon 057, were more abundant among EAs, with Bonferroni-corrected *P* values of 0.03, 0.02, 0.03, 7.88 × 10^−5^, and 0.03, respectively. Another taxon of this phylum, *Peptostreptococcaceae*, showed a higher abundance among AAs (Bonferroni-corrected *P = *2.35 × 10^−4^). The effect sizes of participants’ lifestyle factors on these 13 significant associations are shown in Table S1 and Fig. S3. All of these 13 taxa were also associated with hosts’ percentage of genetic African ancestry at *P < *0.05 (Table 2, Fig. 3B, and Fig. S4). In addition, 10 of these 13 taxa showed a consistent significant differential abundance between AAs and EAs in both sequencing batches (Table S2).

**TABLE 2:** Significantly higher abundance of *Bacteroidetes* and lower abundance of *Actinobacteria* and *Firmicutes* among African-Americans

Taxon	Among all participants (*n* = 1,616)				Genetic African ancestry percentage (*n* = 882)	
	% relative abundance		Coefficient^*a*^	*P* value^*b*^	Coefficient^*a*^	*P* value^*a*^
	European- Americans	African- Americans				
Phylum *Bacteroidetes*						
Family *Porphyromonadaceae*	0.19	0.67	0.38	0.03	0.88	4.81 × 10^−6^
Genus *Porphyromonas*	0.18	0.64	0.37	0.05	0.85	1.91 × 10^−5^
Species *Prevotella denticola*	0.04	0.11	0.49	1.56 × 10^−3^	0.87	1.22 × 10^−5^

Phylum *Actinobacteria*	11.05	10.09	−0.38	6.14 × 10^−8^	−0.46	2.78 × 10^−6^
Family *Micrococcaceae*	5.77	5.47	−0.41	1.81 × 10^−6^	−0.39	8.65 × 10^−4^
Genus *Rothia*	5.77	5.47	−0.42	1.14 × 10^−6^	−0.39	1.04 × 10^−3^
Species *Rothia mucilaginosa*	4.80	4.65	−0.41	1.21 × 10^−5^	−0.35	5.91 × 10^−3^

Phylum *Firmicutes*						
Family *Carnobacteriaceae*	1.35	1.13	−0.22	0.03	−0.26	0.02
Genus *Granulicatella*	1.32	1.09	−0.24	0.02	−0.25	0.03
Species *Granulicatella adiacens*	1.28	1.04	−0.23	0.03	−0.25	0.03
Species *Streptococcus oligofermentans*	0.46	0.22	−0.42	7.88 × 10^−5^	−0.48	8.50 × 10^−4^
Species *Streptococcus* sp. oral taxon 057	9.89	8.85	−0.17	0.03	−0.29	2.77 × 10^−4^
Family *Peptostreptococcaceae*	0.09	0.20	0.41	2.35 × 10^−4^	0.66	5.48 × 10^−6^

a For each sample, centered log-ratio transformation was used to normalize taxon read counts. The associations of taxon abundance with race/African ancestry percentage were evaluated using linear regression analyses.

b Bonferroni-corrected *P* values, adjusted for 25 independent tests.

10.1128/mSystems.00639-19.3 FIG S3 Effect sizes and standard errors of other factors on centered log-ratio-transformed relative abundance of common taxa in combined analyses. Download FIG S3, PDF file, 1.1 MB. Copyright © 2019 Yang et al. 2019 Yang et al. This content is distributed under the terms of the Creative Commons Attribution 4.0 International license.

10.1128/mSystems.00639-19.4 FIG S4 Changes in centered log-ratio-transformed relative abundance of common taxa along with the increase in hosts’ genetic African ancestry. Download FIG S4, PDF file, 0.3 MB. Copyright © 2019 Yang et al. 2019 Yang et al. This content is distributed under the terms of the Creative Commons Attribution 4.0 International license.

10.1128/mSystems.00639-19.7 TABLE S1 Association coefficients of lifestyle factors included in regression analyses of taxon abundance with race. Download Table S1, PDF file, 0.1 MB. Copyright © 2019 Yang et al. 2019 Yang et al. This content is distributed under the terms of the Creative Commons Attribution 4.0 International license.

10.1128/mSystems.00639-19.8 TABLE S2 Associations of common taxon abundance with race stratified by sequencing batch. ^a^For each sample, centered log-ratio transformation was used to normalize taxon read counts. The associations of taxon abundance with race/African ancestry percentage were evaluated using linear regression analyses. Download Table S2, PDF file, 0.04 MB. Copyright © 2019 Yang et al. 2019 Yang et al. This content is distributed under the terms of the Creative Commons Attribution 4.0 International license.

### Rare bacterial taxa with a significant differential prevalence between AAs and EAs.

**FIG 4:**
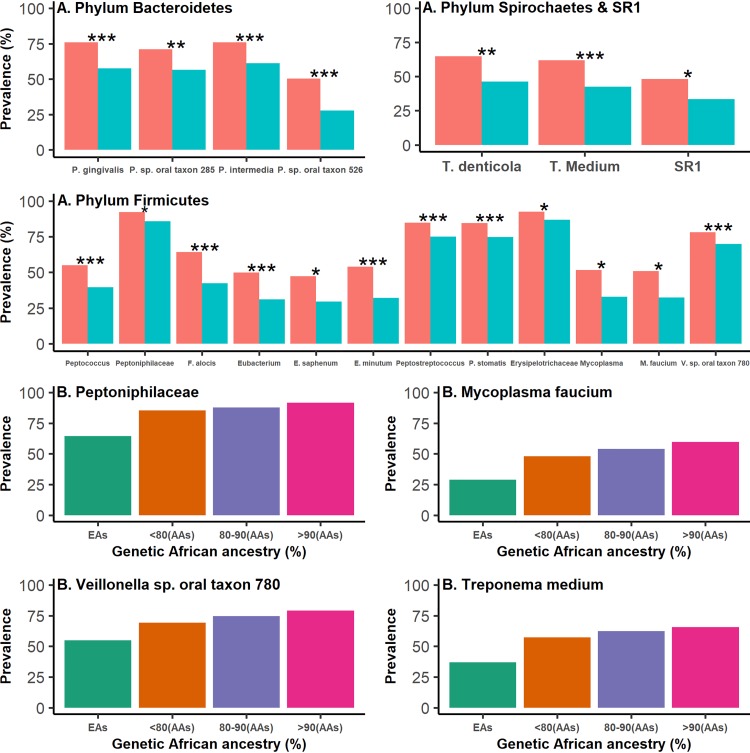
Nineteen rare bacterial taxa showing a significant differential prevalence between AAs and EAs in logistic regression analyses. (A) The associations of taxon prevalence and race were evaluated using logistic regression analyses. *, **, and *** represent Bonferroni-corrected *P* values of <0.05, <0.01, and <0.001, respectively. (B) Increases in prevalence of four rare taxa along with the increase in hosts’ genetic African ancestry. Similar plots for other rare taxa shown in Table 3 are presented in Fig. S6.

In total, 19 rare taxa showed a significant differential prevalence between AAs and EAs in logistic regression analyses, and all of them were more prevalent among AAs (Table 3 and Fig. 4A). Among them, interestingly, four periodontal pathogens, including Porphyromonas gingivalis, Prevotella intermedia, Treponema denticola, and Filifactor alocis, were significantly more prevalent among AAs than among EAs, with Bonferroni-corrected *P* values of 5.23 × 10^−6^, 4.47 × 10^−6^, 1.08 × 10^−3^, and 4.49 × 10^−5^, respectively. Among the remaining 15 taxa, two belonging to *Bacteroidetes*, including *Porphyromonas* sp. oral taxon 285 and *Prevotella* sp. oral taxon 526, were more prevalent among AAs with Bonferroni-corrected *P* values of 8.02 × 10^−3^ and 3.32 × 10^−5^, respectively. In *Firmicutes*, two families, four genera, and five species were more prevalent among AAs. Among them, the species Eubacterium minutum, carried by 32.1% of EAs and 53.9% of AAs, showed the most significant differential prevalence, with Bonferroni-corrected *P = *2.36 × 10^−6^. The phylum *SR1* and a species of the phylum *Spirochaetes*, Treponema medium, showed a significantly higher prevalence among AAs with Bonferroni-corrected *P* values of 0.02 and 6.32 × 10^−4^, respectively. The effect sizes of lifestyle factors, which were adjusted in logistic regression analyses, on these 19 significant associations are presented in Table S3 and Fig. S5. All of these 19 taxa were positively associated with the percentage of genetic African ancestry at *P < *0.05 (Table 3, Fig. 4B, and Fig. S6). In addition, the significant higher prevalence of all these 19 taxa among AAs was also consistently observed in both sequencing batches (Table S4).

**TABLE 3:** Taxa showing a significant different prevalence between African-Americans and European-Americans

Taxon	Among all participants (*n* = 1,616)				Genetic African ancestry percentage (*n* = 882)	
	% prevalence		Coefficient^*a*^	*P* value^*b*^	Coefficient^*a*^	*P* value^*a*^
	European- Americans	African- Americans				
Phylum *Bacteroidetes*						
Species Porphyromonas gingivalis	57.53	76.09	0.75	5.23 × 10^−6^	1.20	4.05 × 10^−7^
Species *Porphyromonas* sp. oral taxon 285	56.45	71.17	0.53	8.02 × 10^−3^	0.80	4.22 × 10^−4^
Species Prevotella intermedia	61.11	76.09	0.79	4.47 × 10^−6^	0.82	4.95 × 10^−4^
Species *Prevotella* sp. oral taxon 526	27.78	50.28	0.70	3.32 × 10^−5^	0.99	9.20 × 10^−6^

Phylum *Firmicutes*						
Genus *Peptococcus*	39.61	55.01	0.66	6.30 × 10^−5^	1.15	2.52 × 10^−7^
Family *Peptoniphilaceae*	85.84	92.16	0.77	0.02	0.86	0.01
Species Filifactor alocis	42.47	64.18	0.67	4.49 × 10^−5^	0.87	8.28 × 10^−5^
Genus *Eubacterium*	31.00	49.72	0.60	7.08 × 10^−4^	0.59	6.49 × 10^−3^
Species Eubacterium saphenum	29.57	47.35	0.51	0.01	0.44	0.05
Species Eubacterium minutum	32.08	53.88	0.75	2.36 × 10^−6^	0.75	6.91 × 10^−4^
Genus *Peptostreptococcus*	75.09	84.78	0.85	9.94 × 10^−6^	1.33	1.35 × 10^−6^
Species Peptostreptococcus stomatis	74.73	84.50	0.87	5.67 × 10^−6^	1.36	7.37 × 10^−7^
Family *Erysipelotrichaceae*	86.74	92.34	0.76	0.03	1.31	7.50 × 10^−4^
Genus *Mycoplasma*	32.80	51.51	0.50	0.02	0.83	1.64 × 10^−4^
Species Mycoplasma faucium	32.44	50.76	0.48	0.03	0.76	5.64 × 10^−4^
Species *Veillonella* sp. oral taxon 780	69.89	78.07	0.74	2.31 × 10^−4^	1.06	4.99 × 10^−5^

Phylum *Spirochaetes*						
Species Treponema denticola	46.24	64.93	0.59	1.08 × 10^−3^	0.82	2.26 × 10^−4^
Species Treponema medium	42.47	61.91	0.61	6.32 × 10^−4^	0.94	2.91 × 10^−5^

Phylum *SR1*	33.33	48.20	0.48	0.02	0.92	1.86 × 10^−5^

a For each taxon, individuals were categorized into carriers and noncarriers according to whether they carried the taxon or not. The associations of taxon prevalence with race/African ancestry percentage were evaluated using logistic regression analyses.

b Bonferroni-corrected *P* values, adjusted for 71 independent tests.

10.1128/mSystems.00639-19.5 FIG S5 Effect sizes and standard errors of other factors on prevalence of rare taxa in combined analyses. Download FIG S5, PDF file, 1.4 MB. Copyright © 2019 Yang et al. 2019 Yang et al. This content is distributed under the terms of the Creative Commons Attribution 4.0 International license.

10.1128/mSystems.00639-19.6 FIG S6 Increases in prevalence of rare taxa along with the increase in hosts’ genetic African ancestry. Download FIG S6, PDF file, 0.3 MB. Copyright © 2019 Yang et al. 2019 Yang et al. This content is distributed under the terms of the Creative Commons Attribution 4.0 International license.

10.1128/mSystems.00639-19.9 TABLE S3 Association coefficients of lifestyle factors included in regression analyses of taxon prevalence with race. Download Table S3, PDF file, 0.1 MB. Copyright © 2019 Yang et al. 2019 Yang et al. This content is distributed under the terms of the Creative Commons Attribution 4.0 International license.

10.1128/mSystems.00639-19.10 TABLE S4 Associations of rare taxon prevalence with race stratified by sequencing batch. ^a^For each taxon, individuals were categorized into carriers and noncarriers according to whether they carried the taxon or not. The associations of taxon prevalence with race/African ancestry percentage were evaluated using logistic regression analyses. Download Table S4, PDF file, 0.1 MB. Copyright © 2019 Yang et al. 2019 Yang et al. This content is distributed under the terms of the Creative Commons Attribution 4.0 International license.

## DISCUSSION

Previous studies have demonstrated the racial differences in the human microbiome, with most studies focusing on the microbiota of the gastrointestinal tract (14, 15), skin (16), and vagina (9, 17). Several studies have also implied the racial differences in the oral microbiome (6, 18, 19). However, these studies had limited sample sizes and the oral microbiome of AAs was not well studied. In the study presented here, we observed significant differences in overall microbial diversity and composition between AAs and EAs and found multiple bacterial taxa, including several preidentified oral pathogens, that showed a significantly different abundance or prevalence between the two racial groups.

In the present study, significant differences in overall microbial composition were observed between AAs and EAs, which were consistent with the results from two previous studies (12, 13). In the first one (13), the saliva microbiome was profiled from 74 native Alaskans, 10 Germans, and 66 Africans. The authors found that Africans had a significant different microbial composition from native Alaskans and Germans. Similarly, in the other study (12) investigating the subgingival microbiome of AAs, EAs, Chinese, and Latinos, a significant difference in overall microbial composition was observed between AAs and EAs. We also found that AAs showed a higher alpha diversity than EAs. However, in both of those studies, AAs showed a lower alpha diversity. This inconsistency could have two potential explanations. First, both of those studies were conducted with very small sample sizes, including 10 to 74 individuals within each group, which is substantially smaller than that (1,058 AAs and 558 EAs) of the present study. Due to the very small sample size, many bacteria of low abundance/prevalence could not be detected, which affected the estimation of microbial diversity. On the other hand, in one study (13), the average sequencing depth was only ∼441 reads per sample, which is much lower than that of the present study (75,021 reads per sample). In the other study (12), microbiomes were profiled using two strategies, i.e., terminal restriction fragment length polymorphism (t-RFLP) and 16S rRNA gene pyrosequencing The latter one, which has higher resolution power, was used for only a portion of the participants, which would have affected the accuracy of taxonomic assignment and then the diversity estimation.

In addition to the difference in overall microbial composition, we found 13 common taxa showing a differential abundance between AAs and EAs. Especially, AAs had a higher abundance of *Bacteroidetes* and a lower abundance of *Actinobacteria* and *Firmicutes*. In the above-mentioned saliva microbiome study (13), several genera showed a significantly different abundance in comparing Africans with native Alaskans and Germans. Several of them, including the higher abundance of *Porphyromonas* and the lower abundance of *Rothia* and *Granulicatella* among Africans, were consistent with results of the present study. In addition, the lower abundance of *Rothia* among AAs was also reported by the above-mentioned subgingival microbiome study (12). No studies have investigated the racial differences of the remaining taxa; hence, a comparison could not be made. Among these 16 taxa, several have been associated with diseases. For example, *Actinobacteria* was reported to be associated with a decreased risk of type 2 diabetes (20). Granulicatella adiacens (21) and Streptococcus oligofermentans (22) were found to be associated with infective endocarditis.

We also found 19 rare taxa that showed a significantly higher prevalence among AAs. Among them, four species, Porphyromonas gingivalis, Prevotella intermedia, Treponema denticola, and Filifactor alocis, have been established to be involved in the pathogenesis of a variety of forms of periodontal diseases (23, 24). Studies have shown a racial disparity in periodontal disease, which is highly correlated with oral bacterial pathogens (25). Several studies have reported that older AAs have more missing and decayed teeth than EAs (26, 27). In addition, data from the National Health and Nutrition Examination Survey (NHANES) showed a 20% greater prevalence of periodontitis (28) and 25% higher rates of dental caries (29) among older AAs (aged 65 years or older) than among older EAs. The differential prevalence of these four oral pathogens may, to some extent, contribute to the disparity of oral health between AAs and EAs. In addition to these oral pathogens, another 15 rare taxa were more prevalent among AAs as well. An earlier study, using 16S rRNA gene cloning and sequencing, found several genera, including *Peptostreptococcus*, associated with periodontitis (30). In addition, one of the species of this genus, Peptostreptococcus stomatis, was observed in peri-implantitis by two recent studies (31, 32). Therefore, the overprevalence of these two taxa might also have contributed to the worse oral health status among AAs than among EAs. However, given that oral hygiene may also contribute to the oral health disparity between the two racial groups but oral hygiene data were not collected from study participants, we could not eliminate the possibility that the enrichment of these periodontal disease-related bacteria in AAs may be attributed to the differences in oral hygiene between AAs and EAs.

To the best of our knowledge, this study is the largest to explore racial differences in the oral microbiome. 16S rRNA gene sequencing was utilized to profile the oral microbiota, which has better resolution than traditional techniques, such as probe-based DNA-DNA hybridization, used in earlier studies. In addition, we adjusted for a variety of covariates among all statistical analyses, making the findings of this study reflect, to the greatest extent, the relationship between oral microbiota and racial affiliation. Further, the availability of genetic data for a portion of study participants made our study the first to evaluate the associations of hosts’ genetic African ancestry with the oral microbiome. A limitation of this study is that it lacks a comprehensive oral health assessment at the baseline examination during the enrollment. In addition, for each of the participants, only one mouth rinse sample was collected; hence, our findings may be impacted by the potential misclassification bias. Further, it is well acknowledged that though 16S rRNA sequencing can provide a stable and accurate resolution for microbiota at the genus level, the species-level profiling was not optimal. Future studies employing the shotgun metagenomic sequencing technology will be needed to fill this gap.

In summary, we found that there were significant differences of overall oral microbiota composition, as well as individual bacterial taxon abundance/prevalence, between AAs and EAs. These results suggest the potential role of oral microbiome in health disparity. The causal mechanisms and factors shaping this difference warrant further investigation in larger sample sizes and with better microbiome profiling techniques.

## MATERIALS AND METHODS

### Study population and data collection.

The SCCS is a prospective study designed to explore health disparities in low-income populations. Details of the study have been described elsewhere (33). Briefly, more than 85,000 adults, aged 40 to 70, were recruited during 2002 to 2009 from 12 states in the southeastern United States, with two-thirds of the participants being AAs. At the enrollment, mouth rinse samples were collected from ∼34,100 participants. Written informed consent was obtained from all study participants. The SCCS was reviewed and approved by Vanderbilt University Medical Center and Meharry Medical College.

During enrollment, the baseline survey was taken by all participants through the filing of a comprehensive questionnaire to gather individuals’ basic information, including age, race/ethnicity, sex, education level, income, lifestyle, anthropometric features, disease history, and so forth. After recruitment, study participants were followed up by using record linkage and mail- or telephone-based surveys. Health-related outcomes were determined from National Death Index mortality records and/or through linkage with state cancer registries.

The present study included participants who provided mouth rinse samples during the study enrollment and were involved in four nested case-control studies to investigate the oral microbiome and incident cases of colorectal cancer, type 2 diabetes, lung cancer, and upper aerodigestive tract cancer. All participants were free of any diseases at the time of mouth rinse sample donation. After excluding participants with a self-reported history of antibiotic usage during the year before biospecimen collection, 1,616 individuals were included in the present study.

### 16S rRNA gene sequencing.

DNA was extracted from mouth rinse samples using Qiagen’s QIAamp DNA kit (Qiagen Inc., Germantown, MD, USA). The NEXTflex 16S V4 Amplicon-Seq kit (Bioo Scientific, Austin, TX, USA) was used to build a library to sequence 253 bp of the V4 domain of the 16S rRNA gene. The data were generated in two batches. For the first batch, 150-bp paired-end sequencing was performed using the Illumina MiSeq 300 at the Vanderbilt Technologies for Advanced Genomics (VANTAGe) Core. For the second batch, 250-bp paired-end sequencing was conducted via the Illumina HiSeq System at BGI Americas (Cambridge, MA, USA). For both batches, each 96-well plate, including an additional negative-control sample and two duplicated quality control (QC) samples, was sequenced. All duplicated samples showed comparable microbial profiles. For example, for the overall microbial richness (alpha diversity measured by Faith’s phylogenetic diversity [PD] index), the coefficient of variability (CV) among the repeated QC samples is 1.7%. For the relative abundance of individual taxa, the median of the Spearman correlation coefficients between the duplicated QC samples is 98.6%.

### Sequencing data processing and quality controls.

For 16S rRNA sequencing data, Sickle (v1.33), BayesHammer, and PANDAseq (v2.10) were used successively to perform low-quality read trimming and removal, sequencing error correction, and paired-end read assembly (34). Then, the merged high-quality reads were processed by Quantitative Insights Into Microbial Ecology (QIIME; v1.9.1). The Human Oral Microbiome Database (HOMD) was used as reference. UCLUST (v1.2.22q) was used for clustering with 97% sequence similarity as the threshold. Those operational taxonomic units (OTUs) observed in fewer than two samples were highly unreliable; hence, they were excluded. Then, the OTU table was summarized to microbial taxon levels.

### Statistical analysis.

For the microbial richness, i.e., alpha diversity, Faith’s PD index was calculated. We first evaluated the associations of participants’ lifestyle factors with alpha diversity through linear regression analyses. Then, the difference of the alpha diversity between AAs and EAs was estimated by the Wilcoxon rank sum test. For the overall microbial composition, i.e., beta diversity, the weighted UniFrac distance, unweighted UniFrac distance, and Bray-Curtis dissimilarity matrices were generated. The beta diversity between AAs and EAs was evaluated through the regression-based kernel method, implemented in MiRKAT (35) (v0.02). We also evaluated whether our data collection was representative enough for both alpha and beta diversity through estimating the changes in alpha and beta diversity along with the increase in number of samples within AAs and EAs, respectively.

For individual taxa, we tested the difference of the relative abundance and/or prevalence at the phylum, family, genus, and species levels between AAs and EAs. First, we investigated the taxa with a relative abundance of >0.10% among AAs, namely, common taxa, including five phyla, 15 families, 16 genera, and 29 species. For each sample, centered log-ratio (clr) transformation was used to normalize taxon read counts. Then, linear regression analysis was conducted with transformed abundance data as outcome and race as independent variables. For those taxa with a relative abundance of ≤0.10% in AAs, namely, rare taxa, we tested their differential prevalence between AAs and EAs via logistic regression. Due to the limited power for the very rare taxa, only those with a prevalence of >30% (with a non-zero read count in >30% of the participants) among AAs were included in the analyses, including four phyla, 19 families, 42 genera, and 102 species.

Genome-wide single nucleotide polymorphism (SNP) array data were available for 397 of the 1,616 individuals, including 324 AAs and 73 EAs, and were used to estimate the percentage of genetic African ancestry for these 397 participants in our previous studies (36–38). Briefly, autosomal common SNPs (minor allele frequency > 0.05) with low linkage disequilibrium (pairwise *r*^2^ < 0.10) were used to estimate the genetic African ancestry, utilizing ADMIXTURE (v1.3.0). We then evaluated the association of the genetic African ancestry percentage with taxon relative abundance or prevalence using linear regression. Among the 73 self-reported EAs, the average African ancestry was only 0.27%. Hence, we included the remaining 485 EAs without genetic data (assuming the African ancestry percentage being 0) in the analyses, resulting in 882 participants in total.

During all of the statistical analyses, we adjusted for the following variants: age, sex, body mass index (BMI), smoking, alcohol consumption, total energy intake, tooth loss, annual household income, state of enrollment, disease status during the first follow-up, and sequencing batch. Among them, BMI and age were treated as continuous variables, and all the other categorical factors were treated as dummy variables, including sex (men and women), smoking (current, former, and never-smoker), alcohol consumption (ever-drinker, never-drinker, and missing), total energy intake (first tertile, second tertile, third tertile, and missing), tooth loss (no teeth lost, loss of 1 to 10 teeth, loss of >10 but not all teeth, loss of all teeth, and missing), annual household income (<$15,000, $15,000 to $50,000, >$50,000, and missing), state of enrollment (12 U.S. states), disease status during the first follow-up (any diseases and no disease), and sequencing batch (first and second batch). Among the factors with missing values, the missing rate is high only for tooth loss (∼34%) and low for all of the others, i.e., ∼5% for total energy intake and ∼1% for annual household income. The microbial taxa at different taxonomic levels are highly correlated. Therefore, Bonferroni correction is too conservative to correct multiple testing. To address this, we used a method described by Galwey (39), implemented in the R package “poolR” (v0.1-0) (https://github.com/ozancinar/poolR/), to evaluate the number of effective tests for common taxa and rare taxa separately. All *P* values were then corrected for multiple testing based on the estimated number of effective tests. For the alpha diversity index, beta diversity matrices, and bacterial taxa that were significantly associated with race, we further conducted stratified analyses by sequencing batch to evaluate the consistency of these associations between batches. All analyses in the present study were carried out using R (v3.3.1) and Python (v2.7.8).

### Data availability.

The 16S rRNA sequencing data used in the present study can be requested through the SCCS online request system (https://www.southerncommunitystudy.org/research-opportunities.html). The R scripts used for statistical analyses of the present study are available in GitHub (https://github.com/YaohuaYangVEC/Codes-for-the-mSystem-paper).
