# Supplementary figures and images for: Racial Differences in the Oral Microbiome: Data from Low-Income Populations of African Ancestry and European Ancestry
Source: mSystems. 2019 Nov 26;4(6):e00639-19. doi: 10.1128/mSystems.00639-19 (PMC6880044; doi:10.1128/mSystems.00639-19)

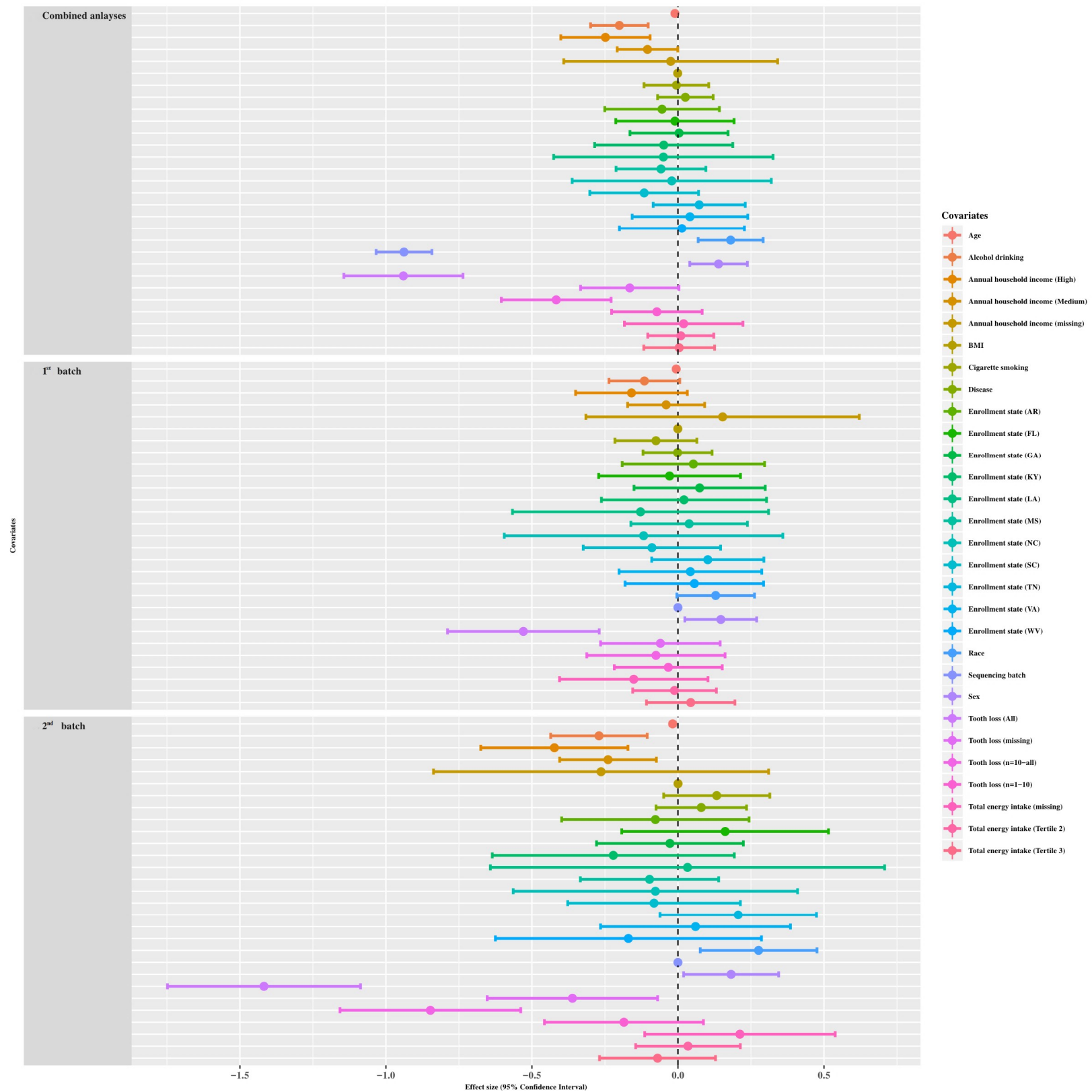

Supplement: FIG S1 [file mSystems.00639-19-sf001.pdf]

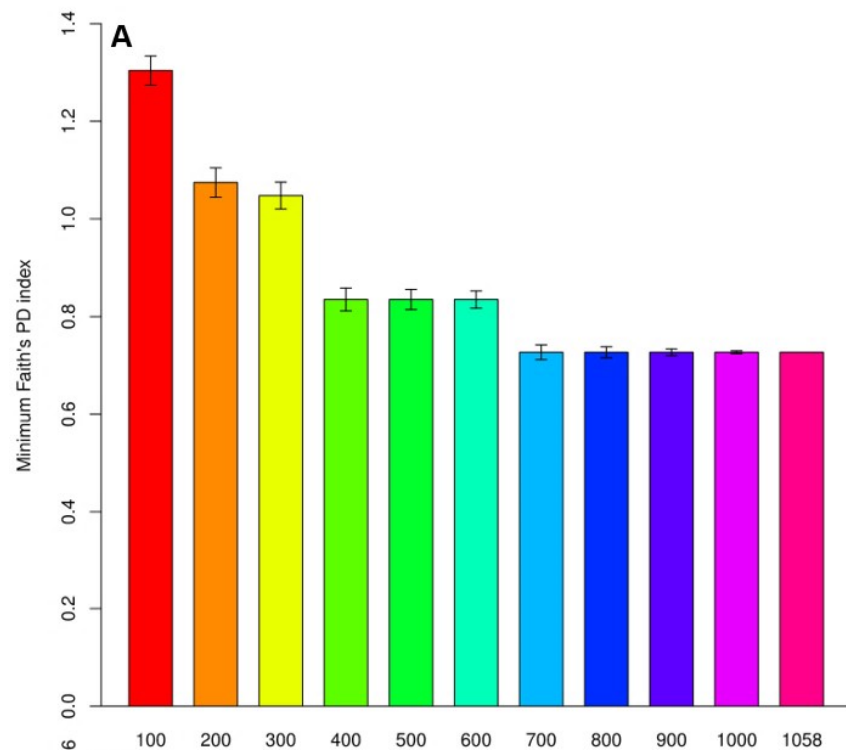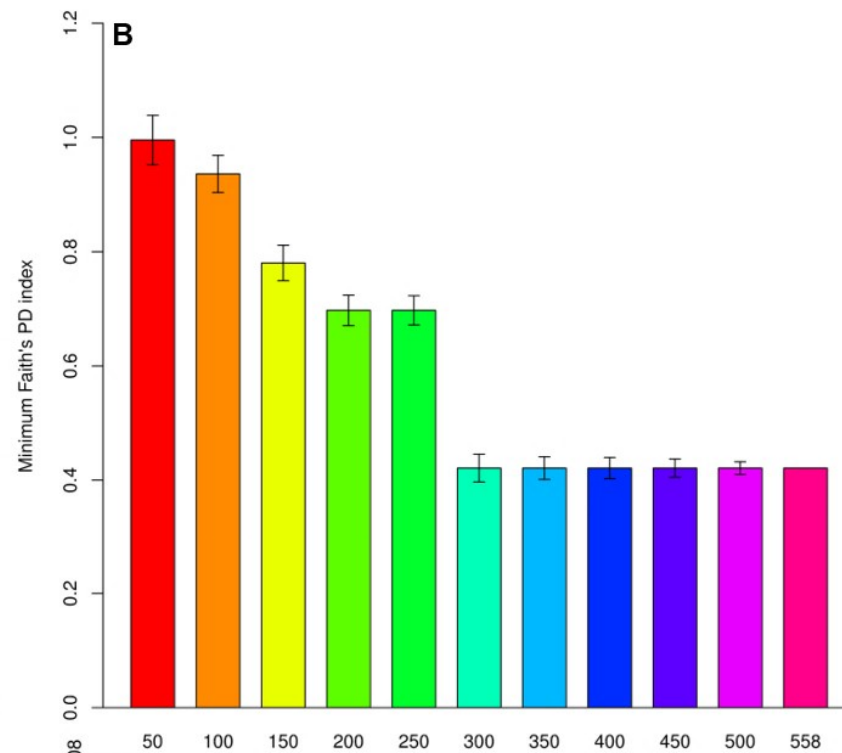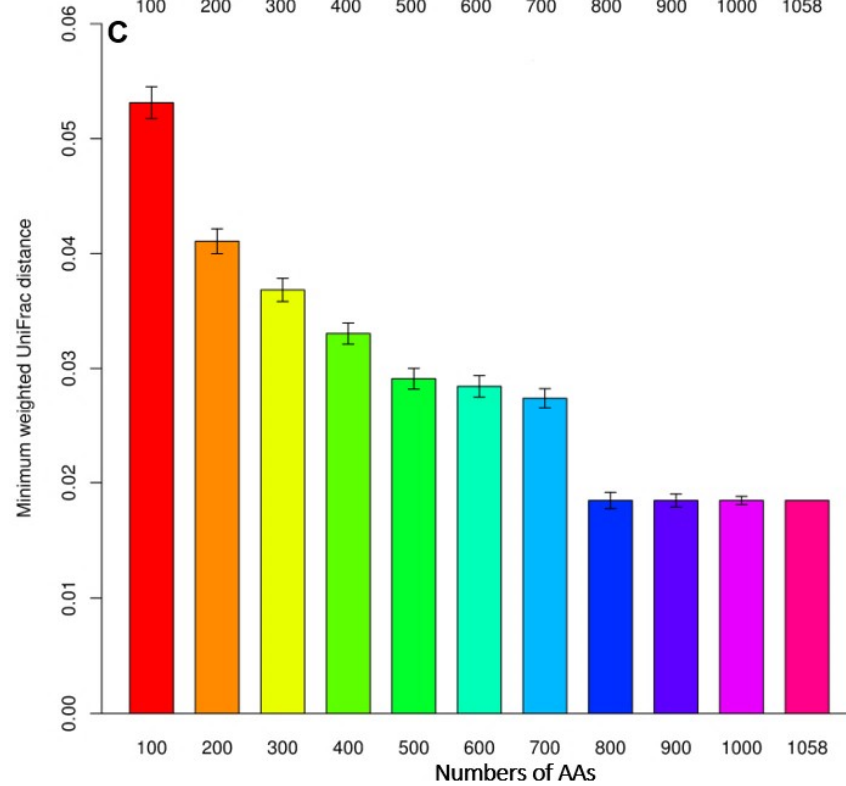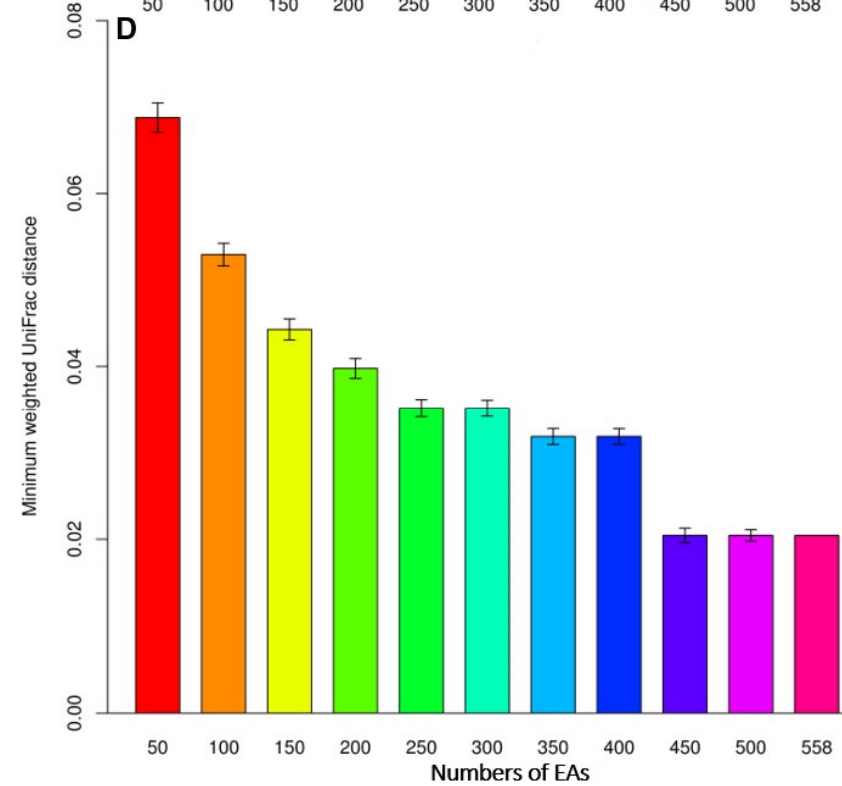

Supplement: FIG S2 [file mSystems.00639-19-sf002.pdf]

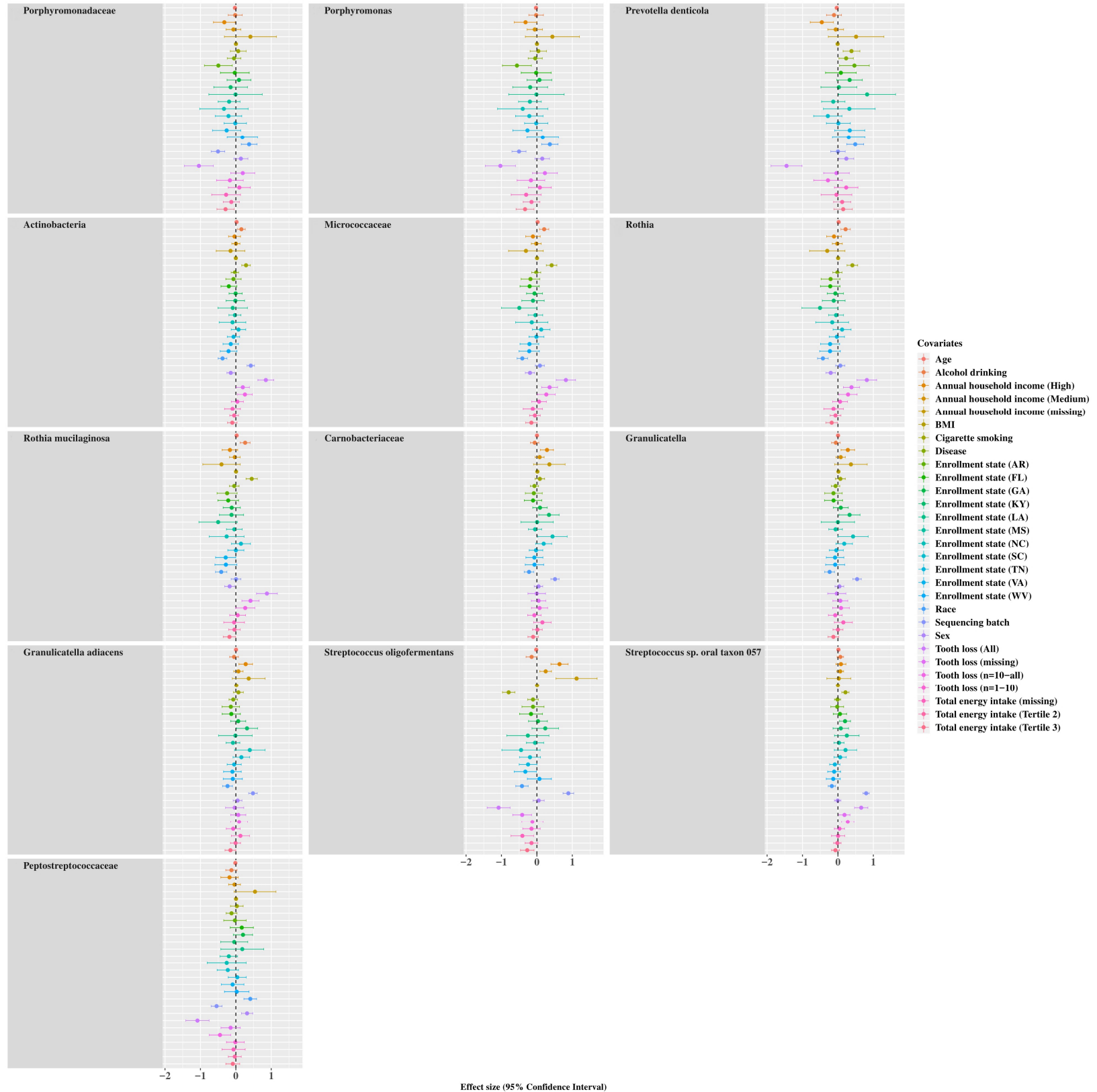

Supplement: FIG S3 [file mSystems.00639-19-sf003.pdf]

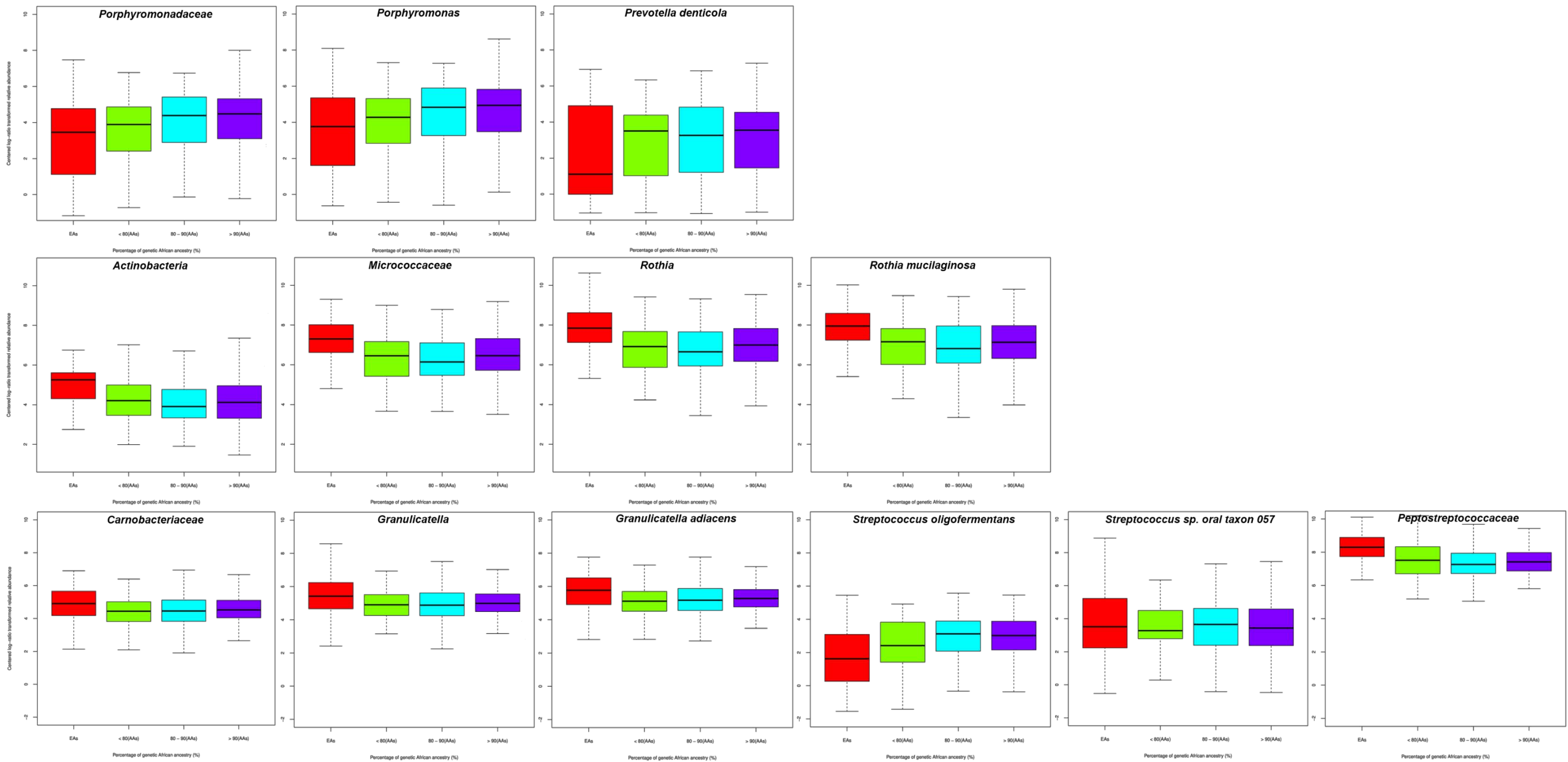

Supplement: FIG S4 [file mSystems.00639-19-sf004.pdf]

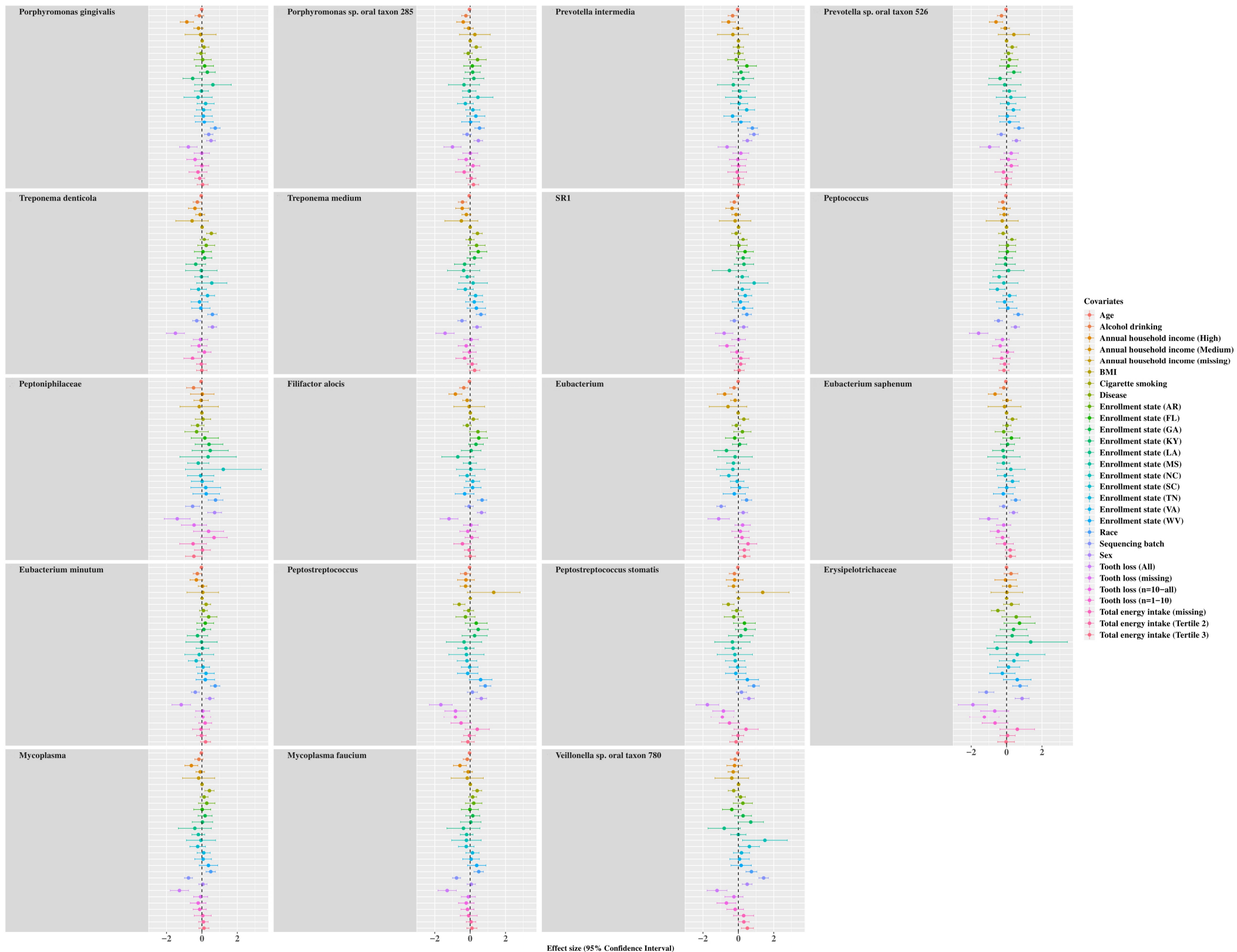

Supplement: FIG S5 [file mSystems.00639-19-sf005.pdf]

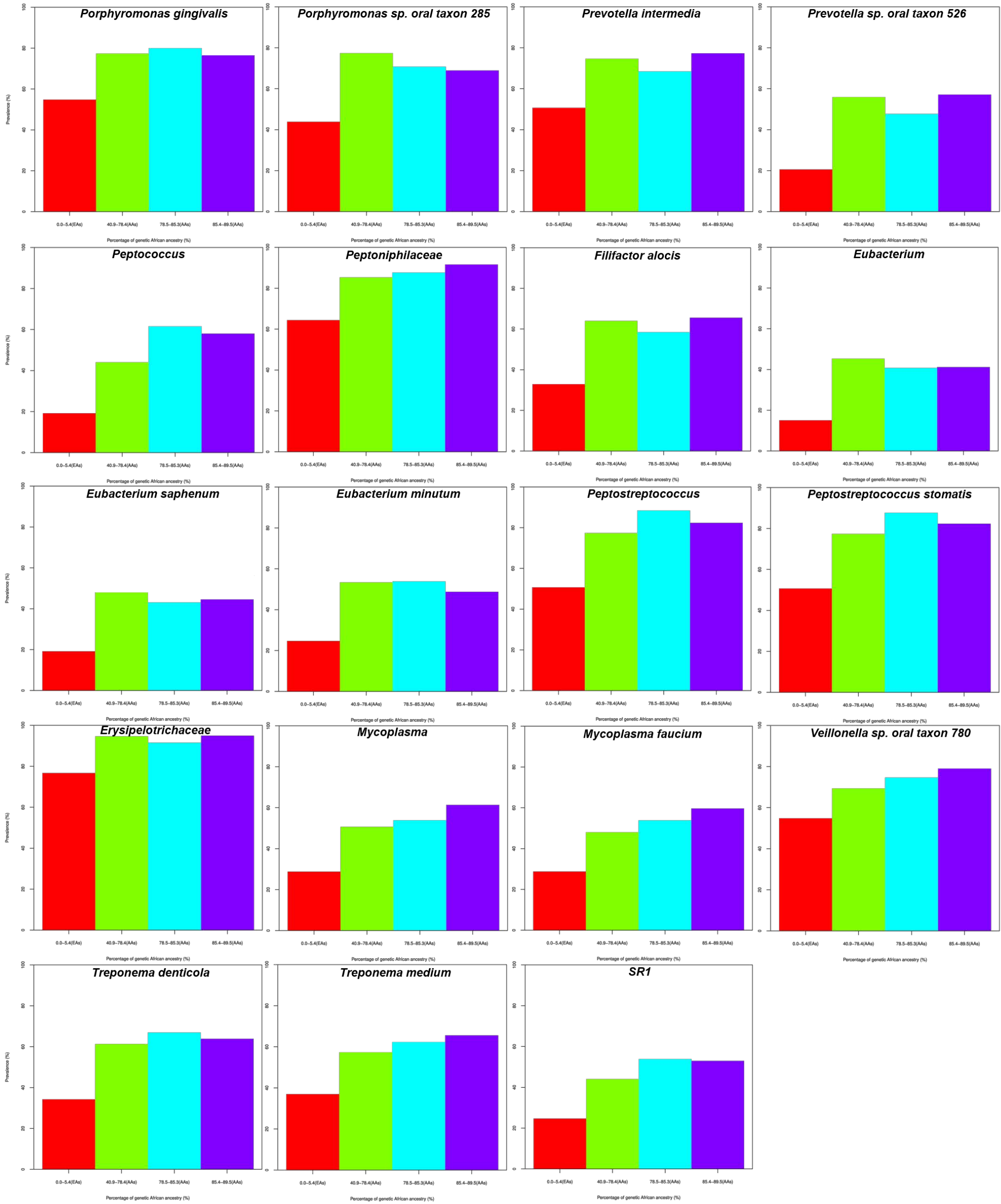

Supplement: FIG S6 [file mSystems.00639-19-sf006.pdf]
