# Supplementary material for: Racial Differences in the Oral Microbiome: Data from Low-Income Populations of African Ancestry and European Ancestry
Source: mSystems. 2019 Nov 26;4(6):e00639-19. doi: 10.1128/mSystems.00639-19 (PMC6880044; doi:10.1128/mSystems.00639-19)
Supplement: TABLE S1 [file mSystems.00639-19-st001.pdf]

Taxa	BMI	Age	Sex	Smoking	Drinking	Total energy intake			Tooth loss (n)				Annual household Income			Enrollment state											Disease	Batch
						T 2	T 3	Missing	1-10	10-all	All	Missing	Medium	High	Missing	AR	FL	GA	KY	LA	MS	NC	SC	TN	VA	WV		
**Phylum *Bacteroidetes***																												
Family *Porphyromonadaceae*	0.00	-0.03	0.14	0.07	-0.02	-0.13	-0.29	-0.28	0.10	-0.17	-1.04	0.19	-0.07	-0.33	0.41	-0.49	-0.03	0.09	-0.15	-0.01	-0.19	-0.34	-0.21	-0.02	-0.26	0.18	-0.05	-0.50
GenusPorphyromonas	0.00	-0.03	0.15	0.04	-0.02	-0.15	-0.33	-0.30	0.08	-0.17	-1.03	0.23	-0.06	-0.32	0.44	-0.56	-0.02	0.07	-0.19	-0.01	-0.20	-0.40	-0.21	-0.02	-0.27	0.16	-0.05	-0.51
Species *Prevotella denticola*	-0.01	-0.03	0.24	0.38	-0.11	0.12	0.15	-0.04	0.23	-0.28	-1.45	-0.04	-0.06	-0.45	0.51	0.47	0.08	0.33	0.03	0.82	-0.13	0.32	-0.29	0.01	0.33	0.30	0.23	0.00
**Phylum *Actinobacteria***	0.00	0.02	-0.14	0.29	0.16	-0.05	-0.10	-0.09	0.04	0.26	0.85	0.20	0.00	-0.04	-0.15	-0.07	-0.20	-0.01	-0.01	-0.09	-0.03	-0.09	0.07	-0.07	-0.14	-0.21	-0.03	0.42
Family *Micrococcaceae*	0.00	0.02	-0.19	0.42	0.21	-0.06	-0.16	-0.12	0.06	0.27	0.82	0.36	-0.02	-0.11	-0.31	-0.18	-0.21	-0.07	-0.11	-0.50	-0.04	-0.14	0.12	-0.01	-0.21	-0.22	-0.02	0.08
Genus *Rothia*	0.00	0.02	-0.20	0.41	0.22	-0.07	-0.18	-0.12	0.06	0.29	0.82	0.38	-0.02	-0.11	-0.30	-0.21	-0.22	-0.07	-0.12	-0.51	-0.05	-0.16	0.12	-0.03	-0.22	-0.22	-0.02	0.07
Species *Rothia mucilaginosa*	0.01	0.02	-0.17	0.45	0.26	-0.04	-0.18	-0.05	0.05	0.26	0.88	0.41	-0.03	-0.17	-0.41	-0.25	-0.22	-0.12	-0.12	-0.50	-0.04	-0.26	0.15	0.00	-0.29	-0.28	-0.04	0.01
**Phylum *Firmicutes***																												
Family *Carnobacteriaceae*	0.01	0.00	0.05	0.08	-0.06	0.02	-0.11	0.16	-0.07	0.07	0.00	0.05	0.08	0.28	0.35	-0.09	-0.11	0.09	0.34	0.01	-0.05	0.44	0.19	-0.02	-0.07	-0.07	-0.07	0.51
Genus *Granulicatella*	0.02	0.00	0.04	0.07	-0.06	0.00	-0.13	0.15	-0.08	0.09	-0.03	0.07	0.08	0.28	0.37	-0.13	-0.12	0.08	0.33	0.00	-0.07	0.43	0.18	-0.05	-0.08	-0.08	-0.07	0.54
Species *Granulicatella adiacens*	0.02	0.01	0.05	0.07	-0.05	-0.01	-0.15	0.13	-0.07	0.09	-0.03	0.07	0.07	0.28	0.36	-0.14	-0.13	0.07	0.31	-0.01	-0.08	0.39	0.16	-0.05	-0.10	-0.09	-0.07	0.48
Species *Streptococcus oligofermentans*	0.01	-0.02	0.05	-0.80	-0.15	-0.15	-0.27	-0.41	-0.15	-0.13	-1.08	-0.42	0.25	0.64	1.12	-0.11	-0.17	0.03	0.24	-0.26	-0.05	-0.45	-0.19	-0.25	-0.33	0.07	-0.11	0.89
Species *Streptococcus sp. oral taxon 057*	0.00	0.02	-0.01	0.21	0.07	-0.02	-0.07	0.01	0.04	0.28	0.66	0.18	0.06	0.09	0.03	-0.02	0.06	0.20	0.08	0.25	0.03	0.21	0.06	-0.09	-0.11	-0.13	-0.01	0.80
Family *Peptostreptococcaceae*	0.01	-0.01	0.32	0.03	-0.13	-0.03	-0.09	-0.06	-0.01	-0.45	-1.08	-0.15	-0.04	-0.18	0.54	-0.03	0.17	0.20	-0.05	0.18	-0.20	-0.26	-0.23	0.04	-0.09	0.02	-0.12	-0.54
