# Supplementary material for: Racial Differences in the Oral Microbiome: Data from Low-Income Populations of African Ancestry and European Ancestry
Source: mSystems. 2019 Nov 26;4(6):e00639-19. doi: 10.1128/mSystems.00639-19 (PMC6880044; doi:10.1128/mSystems.00639-19)
Supplement: TABLE S2 [file mSystems.00639-19-st002.pdf]

Taxa	1st Batch (*N*=956)				2nd Batch (*N*=660)			
	Relative abundance				Relative abundance			
	European-Americans (*N*=402)	African-Americans (*N*=554)	Coefficient [a]	*P* value [a]	European-Americans (*N*=156)	African-Americans (*N*=504)	Coefficient [a]	*P* value [a]
**Phylum *Bacteroidetes***								
Family *Porphyromonadaceae*	0.14%	0.30%	0.16	0.26	0.42%	1.48%	0.88	4.81E-06
Genus *Porphyromonas*	0.14%	0.29%	0.15	0.33	0.40%	1.41%	0.85	1.91E-05
Species *Prevotella denticola*	0.03%	0.10%	0.59	1.07E-04	0.06%	0.15%	0.87	1.22E-05
**Phylum *Actinobacteria***	10.33%	8.02%	-0.35	2.39E-05	13.22%	11.79%	-0.46	2.78E-06
Family *Micrococcaceae*	4.59%	3.70%	-0.43	5.22E-05	8.03%	7.27%	-0.39	8.65E-04
Genus *Rothia*	4.59%	3.70%	-0.44	4.08E-05	8.03%	7.27%	-0.39	1.04E-03
Species *Rothia mucilaginosa*	3.84%	3.07%	-0.45	7.58E-05	6.44%	6.30%	-0.35	5.91E-03
**Phylum *Firmicutes***								
Family *Carnobacteriaceae*	1.29%	0.99%	-0.21	0.02	1.46%	1.25%	-0.26	0.02
Genus *Granulicatella*	1.27%	0.98%	-0.23	0.02	1.40%	1.19%	-0.25	0.03
Species *Granulicatella adiacens*	1.25%	0.95%	-0.23	0.01	1.35%	1.13%	-0.25	0.03
Species *Streptococcus oligofermentans*	0.47%	0.22%	-0.35	1.84E-03	0.42%	0.22%	-0.48	8.50E-04
Species *Streptococcus sp. oral taxon 057*	10.05%	9.84%	-0.13	0.08	8.96%	8.06%	-0.29	2.77E-04
Family *Peptostreptococcaceae*	0.07%	0.11%	0.33	4.19E-03	0.25%	0.44%	0.66	5.48E-06
