# Supplementary material for: Racial Differences in the Oral Microbiome: Data from Low-Income Populations of African Ancestry and European Ancestry
Source: mSystems. 2019 Nov 26;4(6):e00639-19. doi: 10.1128/mSystems.00639-19 (PMC6880044; doi:10.1128/mSystems.00639-19)
Supplement: TABLE S3 [file mSystems.00639-19-st003.pdf]

Taxa	BMI	Age	Sex	Smoking	Drinking	Total energy intake			Tooth loss (n)				Annual household Income			Enrollment state											Disease	Batch
						T 2	T 3	Missing	1-10	10-all	All	Missing	Medium	High	Missing	AR	FL	GA	KY	LA	MS	NC	SC	TN	VA	WV		
**Phylum *Bacteroidetes***																												
Species *Porphyromonas gingivalis*	0.01	-0.02	0.51	0.11	-0.14	-0.12	0.04	-0.22	0.00	-0.39	-0.77	-0.01	-0.19	-0.85	-0.08	0.04	0.15	0.31	-0.52	0.62	-0.03	-0.22	0.21	0.10	0.08	0.13	-0.05	0.38
Species *Porphyromonas sp. oral taxon 285*	0.00	-0.04	0.46	0.35	-0.24	0.06	0.19	-0.34	0.16	-0.23	-1.00	0.01	-0.08	-0.39	0.27	0.42	0.12	0.14	0.21	-0.35	-0.05	0.44	-0.27	0.16	0.32	0.03	-0.11	-0.17
Species *Prevotella intermedia*	0.01	-0.04	0.51	0.00	-0.32	0.01	0.02	-0.07	0.01	-0.03	-0.64	0.14	-0.03	-0.55	-0.31	-0.12	0.48	0.15	0.27	-0.28	0.07	0.11	0.04	0.47	-0.33	0.14	0.03	0.88
Species *Prevotella sp. oral taxon 526*	0.00	-0.03	0.56	0.31	-0.29	0.01	-0.02	-0.17	0.27	0.11	-0.96	0.26	-0.08	-0.60	0.41	0.17	0.10	0.41	-0.37	-0.10	0.15	0.24	0.09	0.38	0.04	0.17	0.11	-0.30
**Phylum *Firmicutes***																												
Genus *Peptococcus*	-0.01	-0.04	0.50	-0.19	-0.22	-0.11	-0.15	-0.28	0.04	-0.36	-1.58	-0.23	-0.14	-0.15	-0.25	0.05	0.04	-0.06	-0.06	0.11	-0.42	-0.16	-0.52	0.17	-0.11	0.08	0.30	-0.46
Family *Peptoniphilaceae*	0.00	-0.05	0.72	0.06	-0.47	0.03	-0.45	-0.50	0.69	0.38	-1.40	-0.45	-0.04	0.02	-0.15	-0.30	0.16	0.40	0.47	0.35	-0.21	1.21	-0.07	0.01	0.21	0.24	-0.24	-0.53
Species *Filifactor alocis*	0.01	-0.04	0.64	0.18	-0.36	-0.06	0.01	-0.44	0.09	-0.13	-1.19	0.04	-0.17	-0.83	-0.05	0.44	0.49	0.33	0.06	-0.70	-0.01	0.03	-0.17	0.15	0.14	-0.31	-0.17	-0.06
Genus *Eubacterium*	0.00	-0.04	0.42	0.29	-0.15	0.23	0.23	-0.12	-0.38	-0.72	-1.25	-0.29	-0.01	-0.63	0.01	-0.13	0.44	0.01	-0.12	-0.19	-0.17	0.15	-0.05	0.42	-0.01	-0.10	-0.02	-0.17
Species *Eubacterium saphenum*	0.00	-0.04	0.39	0.33	-0.16	0.20	0.22	-0.10	-0.23	-0.47	-1.01	-0.16	0.02	-0.65	-0.12	-0.16	0.27	0.06	-0.20	-0.15	-0.17	0.24	-0.07	0.33	0.01	-0.19	0.02	-0.17
Species *Eubacterium minutum*	0.01	-0.03	0.45	0.24	-0.26	-0.03	0.21	-0.05	0.18	0.06	-1.17	0.04	0.03	-0.31	0.05	0.38	0.19	0.11	-0.25	-0.02	0.03	-0.15	-0.32	0.06	0.23	0.19	0.09	-0.37
Genus *Peptostreptococcus*	0.01	-0.03	0.63	-0.62	-0.26	-0.04	-0.13	0.40	-0.51	-0.83	-1.66	-0.83	-0.26	-0.24	1.33	-0.26	0.35	0.45	0.25	-0.34	-0.24	-0.21	-0.18	-0.03	-0.14	0.59	-0.08	0.13
Species *Peptostreptococcus stomatis*	0.01	-0.03	0.60	-0.57	-0.22	-0.02	-0.13	0.44	-0.50	-0.90	-1.75	-0.85	-0.28	-0.21	1.38	-0.26	0.34	0.40	0.14	-0.34	-0.30	-0.20	-0.18	-0.04	-0.15	0.51	-0.09	0.18
Family *Erysipelotrichaceae*	0.00	-0.01	0.88	0.28	0.25	0.06	-0.03	0.60	-0.65	-1.26	-1.90	-0.67	0.18	-0.06	14.71	0.55	0.73	0.39	0.32	1.36	-0.54	0.61	0.41	0.11	-0.24	0.60	-0.50	-1.15
Genus *Mycoplasma*	0.01	-0.03	0.05	0.43	-0.17	0.08	0.11	0.05	-0.12	-0.21	-1.27	-0.07	-0.10	-0.60	-0.19	0.27	0.02	0.18	0.03	-0.40	-0.20	-0.05	-0.24	0.10	0.07	0.37	0.12	-0.76
Species *Mycoplasma faucium*	0.01	-0.03	0.06	0.40	-0.16	0.05	0.10	-0.08	-0.15	-0.22	-1.30	-0.10	-0.11	-0.58	-0.16	0.20	-0.02	0.15	0.04	-0.38	-0.20	-0.21	-0.22	0.13	0.05	0.37	0.14	-0.77
Species *Veillonella sp. oral taxon 780*	0.01	-0.04	0.50	-0.26	-0.18	0.31	0.51	0.30	-0.18	-0.67	-1.21	-0.25	-0.29	-0.22	-0.37	0.25	-0.37	0.27	0.70	-0.80	0.00	1.50	0.62	0.18	0.07	0.16	0.13	1.43
**Phylum *Spirochaetes***																												
Species *Treponema denticola*	0.01	-0.04	0.60	0.53	-0.26	-0.03	-0.01	-0.53	0.14	-0.16	-1.50	-0.08	-0.10	-0.39	-0.56	0.24	0.06	0.15	-0.35	-0.03	-0.03	0.56	-0.19	0.32	-0.13	-0.06	0.14	-0.29
Species *Treponema medium*	0.00	-0.05	0.39	0.41	-0.43	0.11	0.25	-0.31	-0.03	-0.22	-1.42	0.05	-0.21	-0.45	-0.50	0.36	0.46	0.24	-0.31	-0.37	-0.16	0.16	-0.27	0.31	0.23	0.35	-0.01	-0.46
**Phylum *SR1***	0.01	-0.03	0.30	-0.12	-0.23	0.13	0.04	0.14	-0.08	-0.65	-0.80	0.01	-0.13	-0.36	-0.18	0.03	0.38	0.27	0.32	-0.51	0.22	0.89	0.22	0.39	0.12	0.30	0.27	-0.23
