# Supplementary material for: Racial Differences in the Oral Microbiome: Data from Low-Income Populations of African Ancestry and European Ancestry
Source: mSystems. 2019 Nov 26;4(6):e00639-19. doi: 10.1128/mSystems.00639-19 (PMC6880044; doi:10.1128/mSystems.00639-19)
Supplement: TABLE S4 [file mSystems.00639-19-st004.pdf]

Taxa	1st Batch (*N*=956)				2nd Batch (*N*=660)			
	Prevalence				Prevalence			
	European-Americans (*N*=402)	African-Americans (*N*=554)	Coefficient [a]	*P* value [a]	European-Americans (*N*=156)	African-Americans (*N*=504)	Coefficient [a]	*P* value [a]
**Phylum *Bacteroidetes***								
Species *Porphyromonas gingivalis*	61.94%	79.96%	0.53	3.37E-03	46.15%	71.83%	1.11	3.47E-06
Species *Porphyromonas sp. oral taxon 285*	56.97%	72.20%	0.41	0.02	55.13%	70.04%	0.67	5.88E-03
Species *Prevotella intermedia*	68.16%	84.84%	0.58	3.37E-03	42.95%	66.47%	1.16	1.73E-06
Species *Prevotella sp. oral taxon 526*	29.35%	49.28%	0.50	4.38E-03	23.72%	51.39%	1.12	1.25E-05
**Phylum *Firmicutes***								
Genus *Peptococcus*	40.55%	50.72%	0.48	4.64E-03	37.18%	59.72%	1.04	3.01E-05
Family *Peptoniphilaceae*	85.82%	90.61%	0.50	0.05	85.90%	93.85%	1.49	8.72E-04
Species *Filifactor alocis*	44.53%	65.70%	0.47	5.53E-03	37.18%	62.50%	1.05	1.68E-05
Genus *Eubacterium*	32.34%	50.90%	0.50	3.30E-03	27.56%	48.41%	0.74	3.58E-03
Species *Eubacterium saphenum*	30.85%	48.38%	0.43	0.01	26.28%	46.23%	0.63	0.01
Species *Eubacterium minutum*	31.84%	52.17%	0.71	2.94E-05	32.69%	55.75%	0.91	1.84E-04
Genus *Peptostreptococcus*	77.36%	85.20%	0.61	3.82E-03	69.23%	84.33%	1.08	1.20E-04
Species *Peptostreptococcus stomatis*	77.36%	85.20%	0.61	3.82E-03	67.95%	83.73%	1.09	9.31E-05
Family *Erysipelotrichaceae*	85.57%	88.81%	0.60	0.02	89.74%	96.23%	1.42	3.79E-03
Genus *Mycoplasma*	29.85%	45.49%	0.43	0.01	40.38%	58.13%	0.69	4.51E-03
Species *Mycoplasma faucium*	29.60%	44.40%	0.40	0.02	39.74%	57.74%	0.70	3.92E-03
Species *Veillonella sp. oral taxon 780*	79.60%	88.99%	0.61	6.43E-03	44.87%	66.07%	0.80	8.18E-04
**Phylum *Spirochaetes***								
Species *Treponema denticola*	47.51%	64.44%	0.44	9.47E-03	42.95%	65.48%	0.85	7.36E-04
Species *Treponema medium*	41.29%	61.55%	0.51	3.33E-03	45.51%	62.30%	0.85	6.29E-04
**Phylum** *SR1*	32.59%	48.01%	0.46	5.96E-03	35.26%	48.41%	0.65	7.91E-03
